# Gut microbiome modulation can improve cognitive function in older adults: a systematic review and meta-analysis

**DOI:** 10.1093/ageing/afag287

**Published:** 2026-09-29

**Authors:** Benjamin Birns, Sabiha Murshed, Mary Ni Lochlainn

**Affiliations:** GKT School of Medical Education, King's College London, London, UK; The University of Edinburgh School of Physics and Astronomy - United Kingdom Centre for Astrobiology (UKCA), Edinburgh, Scotland, UK; GKT School of Medical Education, King's College London, London, UK; Department of Twin Research and Genetic Epidemiology, King's College London - Centre for Ageing Resilience in a Changing Environment, London, UK

**Keywords:** gut microbiome, cognition, dementia, nutritional supplements, probiotics, older people, systematic review

## Abstract

**Background:**

Age-related cognitive decline and dementia exert increasing pressure on healthcare systems. Gut microbiome modulation may offer a novel approach to improving cognitive health.

**Objective:**

To establish the effect of gut microbiome modulation on cognitive function in older adults.

**Methods:**

A systematic search was conducted of PubMed, Cochrane Library, Medline and Embase (OVID) to identify randomised controlled trials (RCTs) and observational studies assessing cognitive outcomes in adults aged ≥60. Interventions aimed at modifying the gut microbiome (prebiotics, probiotics, synbiotics) were included. Studies involving faecal transplants or comparing modifiers to one another instead of to placebo were excluded. Changes in cognitive function were measured by neuropsychological assessments and standardised cognitive tests (e.g. MMSE, MoCA).

**Results:**

Fourteen studies met inclusion criteria (13 RCTs, 1 interventional study), consisting of 10 probiotic, 2 prebiotic and 2 synbiotic trials. Participants included both healthy older adults and those with cognitive impairment. Nine studies reported statistically significant improvements in at least one cognitive domain, particularly global cognition, memory, attention and executive function. Meta-analysis of a sub-group resulted in a statistically significant (MD = 1.28; 95% CI = 0.15–2.40; *P* < 0.05) improvement in global cognition scores after supplementation.

**Conclusions:**

Gut microbiome modulation through probiotic, prebiotic and synbiotic supplementation can improve cognition in older adults. Whilst heterogeneity and paucity of high-quality literature exists, the overall evidence is positive. Gut microbiome modulation therefore holds huge promise as a viable strategy to support cognitive health in older people, particularly considering the interventions are mostly already commercially available and low-risk.

## Key points

- Gut microbiome modulation via probiotic, prebiotic, synbiotic intervention is associated with improvements in cognitive outcomes.
- Gut microbiome modulation represents a promising, low-risk and affordable approach to support cognitive ageing.
- Gut microbiome modulation evidence was strongest for global cognition, memory and executive function.
- Cognitive benefits were more consistently observed in cognitively healthy older adults and those with mild cognitive impairment.

## Introduction

The global population is ageing at an accelerating rate [1] with the number of people aged ≥60 expected to nearly double by 2050. This demographic shift will substantially increase the prevalence of age-related conditions [2], particularly cognitive decline, which is a key aspect of frailty and associated with a loss of independence [3]. Neurodegenerative diseases, such as dementia, represent the greatest deterioration of cognition and are accompanied by greater care needs [4], placing significant strain on health services [5]. Innovative and effective approaches to support cognitive health in older adults is therefore a public health priority.

The gut microbiome, with its influence on systemic inflammation [6], neurochemical pathways [7] and immune regulation [8], offers a potential pathway for intervention. The gut microbiome is a diverse microbial community in the gastrointestinal tract that has a symbiotic relationship with the human host [9], supporting health directly through digestion, nutrient synthesis and immunoregulation [10]. However, it is increasingly apparent that the gut microbiome influences aspects of health beyond the gastrointestinal system. Microbial metabolites, including short-chain fatty acids (e.g. butyrate) and neurotransmitters (e.g. serotonin, dopamine, acetylcholine and gamma-aminbutyric acid (GABA) produced by commensal bacteria from genera *Bifidobacterium* and *Lactobacillus*), influence the central nervous system, endocrine pathways and the immune system [11]. Bidirectional communication between the gut microbiome and the brain occurs through neural pathways, including the enteric nervous system via the vagus nerve, as well as neuroendocrine mechanisms, such as the hypothalamic–pituitary–adrenal axis, to influence mood, cognition and behaviour [12]. It is through these various channels that the gut microbiome interacts with the brain and forms, what is commonly referred to as, the gut-brain axis [13].

A growing body of evidence suggests that alterations to the gut microbiome may contribute to the development of neurodegenerative diseases, such as Alzheimer's and Parkinson's disease [14, 15]. Hence, the question arises whether interventions targeting the gut microbiome could positively impact cognitive function in older people. Whilst the gut microbiome acts as a balanced ecosystem of microbial species, it may be modulated through diet/supplementation, such as probiotics (live bacteria), prebiotics (a non-digestible food ingredient that promotes the growth of beneficial microorganisms in the intestines) and synbiotics (a combination of probiotics and prebiotics) [16]. These can be incorporated as part of a balanced diet with raw vegetables, fermented foods or dairy products, and/or via supplementation [17].

This systematic review and meta-analysis synthesise current evidence to evaluate gut microbiome-targeted interventions for improving cognitive health in older adults.

## Methods

This systematic review followed the Preferred Reporting Items for Systematic Review and Meta-Analysis (PRISMA) guidelines. The protocol was registered with the International Prospective Register of Systematic Reviews (CRD420251029045).

### Search strategy and study selection

A systematic search was conducted by two independent reviewers (SM & KS) of PubMed, Cochrane Library, Medline and Embase (OVID) from inception to 19 October 2024, without language restriction. This was repeated and updated on 13 April 2025 to identify any additional or recently published articles. The full search terms and strategies, alongside inclusion and exclusion criteria, are included in Appendices (see Supplementary material).

All search results were imported to Rayyan.ai [18] for deduplication, after which titles and abstracts were manually screened by SM and KS to assess eligibility for full-text screening. The reviewers screened articles independently to reduce any potential selection bias. Following that, a 25% random sample was re-screened by BB to assess inter-reviewer consistency. Full texts were then screened by BB and SM, independently, and text references were additionally reviewed to identify missed studies by initial search strategies. Resolution of screening conflicts was achieved by consensus. No large-language model or other ‘artificial intelligence’ screening was used.

### Data extraction and risk of bias

Data was extracted by BB and checked for accuracy by SM. Details extracted included the type of intervention (probiotics, prebiotics or synbiotics), dosage of intervention, comparator type (placebo or standard care), methods of outcome assessments and the reported results. The risk of bias was assessed by SM using the Cochrane RoB2 tool for randomised control trials (RCTs) [19], which systematically evaluates reliability and credibility of study findings across five domains relevant to trial design, conduct and reporting. For assessment of observational studies, ROBINS-I tool [20] was used.

### Statistical analyses

Where RCTs met inclusion criteria and shared outcome measures, quantitative statistical comparisons were performed as a meta-analysis, using Cochrane’s Review Manager 5.4.1 software [21]. Inverse-variance methods were applied within a random-effects model for anticipated clinical and methodological variability in order to assess the statistical significance of continuous data recorded as mean values. Authors were contacted to obtain missing data; in the absence of a response, standard deviations of mean differences (MD) were derived from standard errors or *P*-values from t-tests or a conservative correlation coefficient of 0.7 was used in calculations. To measure effect size, standardised mean differences (SMD) were additionally calculated using pooled standard deviations. Heterogeneity was assessed by the overlap of 95% confidence intervals (CI) and quantified as I^2^ values.

Sensitivity analyses were conducted to evaluate the robustness of the findings by excluding articles judged to be above low risk of bias or removing outliers and comparing to initial results. Subgroup analyses were used to assess the impact of key methodological factors, such as intervention type and the exclusion of participants with advanced baseline cognitive impairment. Any substantial changes in effect estimates were reported and discussed.

The certainty of evidence for each outcome was assessed using the Grading of Recommendations Assessment, Development, and Evaluation (GRADE) tool, which classifies the overall quality of evidence as either high, moderate, low or very low across five domains: risk of bias, inconsistency, indirectness, imprecision and publication bias [22].

## Results

### Study selection and characteristics

Literature searches identified 3423 articles and after duplicates were removed, 3092 articles were screened on title and abstract, where 60 articles were deemed eligible for full text screening. Of these, 46 were excluded, resulting in 14 studies being included (Figure 1). Inclusion and exclusion decisions were concordant for 100% of screened articles. Thirteen of the studies were RCTs of which 11 were double-blinded and 1 was an interventional non-randomised study [23]. Characteristics of included studies are summarised in Table 1.

**Figure 1:**
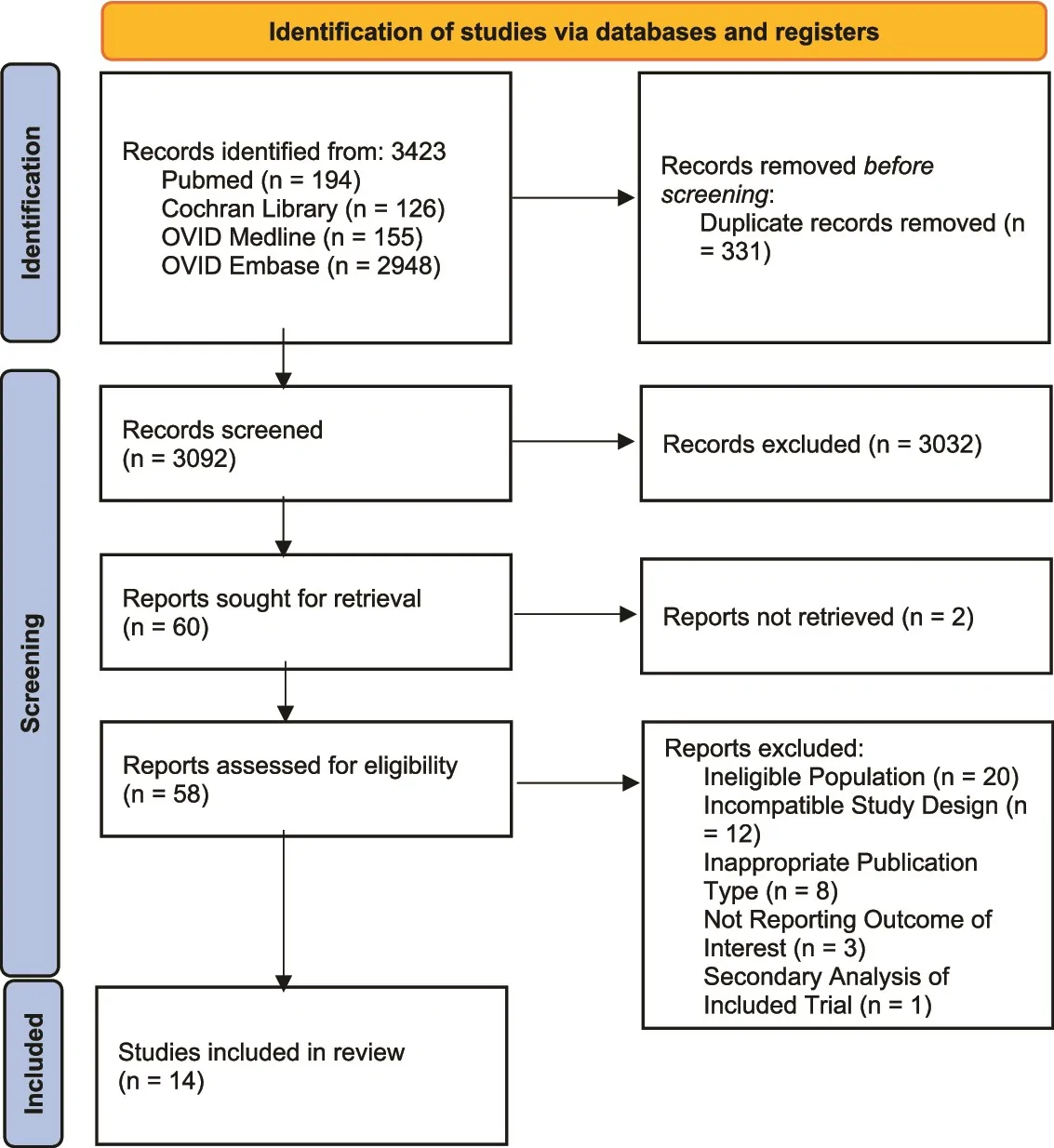
PRISMA 2020 flow diagram.

**Table 1:** Study characteristics.

Study	Baseline control group characteristics	Baseline intervention group characteristics	Intervention	Comparator	Outcome
Akbari (2016) DB-RCT of outpatients in Iran	*n* = 30 (six male) mean (SEM) age = 82 (1.69) mean (SEM) MMSE = 8.47 (1.10)	*n* = 30 (6 male) mean (SEM) age = 77.67 (2.62) mean (SEM) MMSE) = 8.67 (1.44)	*Lactobacillus acidophilus*, *Lactobacillus casei*, *Bifidobacterium bifidum*, and *Lactobacillus fermentum*, 2x10^9^ CFU/g for 12 weeks	Milk	MMSE: Significant improvement, *P* < 0.001
Buigues (2016) DB-RCTS in nursing home in Spain	*n* = 22 (6 male) mean (SEM) age = 73.4 (1.8) mean (SEM) MMSE = 26.5 (3.1)	*n* = 28 (9 male) mean (SEM) age = 74.2 (1.6) mean (SEM) MMSE = 26.1 (2.2)	Inulin 3375 mg and FOS 3488 mg of per level measuring spoon (of 7.5 grams) for 13 weeks	Maltodextrin	MMSE: No significant difference, *P* = 0.85
Agahi (2018) DB-RCT in welfare organisations in Iran	*n* = 23 (10 male) mean (SD) age = 80.57 (1.79) mean (SD) TYM = 14.35 (2.27)	*n* = 25 (7 male) mean (SD) age = 79.7 (1.72) mean (SD) TYM = 14.64 (1.71)	*L. fermentum*, *Lactobacillus plantarum*, *Bifidobacterium lactis*, *L. acidophilus*, *B. bifidum*, and *Bifidobacterium longum*, two capsules of 3 × 10^9^ CFU for 12 weeks	500 mg Maltodextrin	TYM: No significant difference, *P* = 0.82
Kim (2021) DB-RCT in community setting in Republic of Korea	*n* = 26 (16 male) mean (SD) age = 72.00 (3.36) mean (SD) MMSE = 26.62 (2.25)	*n* = 27 (10 male) mean (SD) age = 71.11 (5.02) mean (SD) MMSE = 26.93 (2.42)	*B. bifidum* BGN4 and *B. longum* BORI, 1 × 10^9^ CFU/day for 12 weeks	Soybean Oil	CERAD-K: Mental flexibility significantly improved, *P* < 0.05 No significant changes in language, memory, visuospatial or other executive functions
Wang (202)1 DB-RCT in hospital in China	*n* = 61 (24 male) mean (SD) age = 70.64 (6.93) mean (SD) MMSE = 25.39 (3.54)	*n* = 59 (29 male) mean (SD) age = 69.56 (5.67) mean (SD) MMSE =25.19 (3.37)	*B. longum*, *L. acidophilus* and *Enterococcus* *faecalis*, twice daily 0.84 g capsule of 1× 10^7^ CFU/capsule from admission until 7 days post-surgery	Lactose	MMSE: significant difference, *P* = 0.020 POCI Incidence: significantly lower, *P* = 0.046
Asaoka (2022) DB-RCT of outpatients in Japan	*n* = 60 (25 male) mean (SD) age = 78.9 (4.3) mean (SD) MMSE = 24.6 (1.2) ADAS = 7.52 (3.06)	*n* = 55 (26 male) mean (SD) age = 77.2 (5.8) mean (SD) MMSE =24.5 (1.2) ADAS 7.98 (2.89)	*Bifidobacterium breve* MCC1274, 2 × 10^10^ CFU for 24 weeks	Maize Starch	ADAS-Jcog no significant intergroup differences; significant improvement was in orientation, *P* = 0.021). MMSE no significant differences; significant intergroup difference was observed in the sub-domain score ‘repetition’ at week 16 (*P* = 0.033)
Handajani (2022) RCT in community setting with care support in Indonesia (two intervention groups)	*n* = 28 (9 male) age = 21 (27.0%) over 65 7 (25.0%) 60–65 CDT = 0.86 WLMIR 1 = 2.64 WLMIR 3 = 5.18 Learning Process = 2.54 Verbal Fluency = 11.71	*n* = 33 (10 male) age = 26 (77.8%) over 65 7 (21.2%) 60–65 CDT 0.61 WLMIR 1 2.21 WLMIR 3 3.88 Learning Process 1.67 Verbal Fluency 9.97	*Limosilactobacillus fermentum* from Tempeh A, Intervention A: 100 ml of 1 × 10^8^ CFU/ml for 12 weeks Intervention B: 100 ml of 1 × 10^7^ CFU/ml for 12 weeks	Maltodextrin	Intervention A: significant improvement in memory and learning process, *P* < 0.05 Intervention B: significant improvement in executive function and visuospatial, memory and verbal fluency, p < 0.05 Control: significant improvement in memory, p < 0.05
		*n* = 32 (14 male) age = 25 (78.1%) over 65 7 (21.9%) 60–65 CDT = 0.69 WLMIR 1 = 2.31 WLMIR 3 = 4.63 Learning Process = 2.31 Verbal Fluency = 11.47			
Sakurai (2022) DB-RCT in the community in Japan	*n* = 39 (18 male) mean (SD) age = 76.9 (4.9) mean (SD) Composite Memory = 92.8 (8.0)	*n* = 39 (18 male) mean (SD) age = 76.8 (4.6) mean (SD) Composite Memory = 91.3 (9.7)	*Lactiplantibacillus plantarum* OLL2712, 1 g of 5 × 10^9^ CFU for 12 weeks	1 g of TK-16AG dextrin	Composite Memory (Visual + Verbal): no significant difference, *P* = 0.058 Sud-domain visual memory significantly improved, *P* = 0.044 (*P* < 0.05)
Fei (2023) RCT in hospitals in China	*n* = 21 (11 male) mean (SD) age = 75.3 (9.75) mean (SD) MMSE = 20.85 (2.25) MoCA = 19.80 (1.15)	*n* = 21 (10 male) mean (SD) age = 76.4 (9.61) mean (SD) MMSE = 21.75 (2.57) MoCA = 19.80 (1.85)	*L. plantarum* BioF-228, *Lactococcus lactis* BioF-224, *B. lactis* CP-9, *Lactobacillus rhamnosus* Bv-77, *Lactobacillus johnsonii* MH-68, *Lactobacillus paracasei* MP137, *Lactobacillus salivarius* AP-32, *L. acidophilus* TYCA06, *L. lactis* LY-66, *B. lactis* HNO19, *L. rhamnosus* HNO01, *L. paracasei* GL-156, *Bifidobacterium animalis* BB-115, *L. casei* CS-773, *Lactobacillus reuteri* TSR332, *L. fermentum* TSF331, *Bifidobacterium infantis* BLI-02, and *L. plantarum* CN2018, 2 g of 2x10^10^ CFU/g for 12 weeks	Starch	MMSE significantly improved,*P* < 0.001; sub-domains attention and calculation(*P* < 0.001) and recall (*P* ≤ 0.001) scores. MoCA significantly improved, *P* = 0.00; sub-domains visuospatial and executive (*P* < 0.001) and recall (*P* < 0.001) scores improved.
Shi (2023) DB-RCT in the community in China	*n* = 30 (13 male) mean (SD) age = 64.5 (3.79) mean (SD) MoCA = 22.97 (2.43) RBANS = 186.93 (15.62)	n = 30 (12 male) mean (SD) age = 64.1 (3.4) mean (SD) MoCA = 23.03 (2.50) RBANS = 186.90 (17.22)	*B. longum* BB68S, 5 × 10^10^ CFU for 8 weeks	Maltodextrin	RBANS: significantly improved, *P* < 0.0001; sub-domains significant improvement in immediate memory, visuospatial/constructional, attention and delayed memory, *P* < 0.0001
Lazou-Ahren (2024) DB-RCT in the community in Sweden (2 intervention groups)	*n* = 22 (16 male) mean (SD) age = 73.1 (2.1) mean (SD) TMT-A = 45.75 (10.21) TMT-B = 84.36 (44.08)	*n* = 22 (11 male) mean (SD) age = 73.2 (1.8) mean (SD) TMT-A = 46.55 (10.21) TMT-B = 66.41 (25.48)	*Lactiplantibacillus plantarum* HEAL9 with/without a mixture of freeze-dried blackberries and black currants, 1x10^10^ CFU/sachet with/without 6 g freeze-dried powder, consisting of 3 g of each berry, derived from 43 g of fresh berries for 4 weeks	Maltodextrin	No significant difference between groups for TMT-A or TMT-B score
		*n* = 22 (16 male) mean (SD) age = 73.2 (1.9) mean (SD) TMT-A = 50.00 (37.12) TMT-B = 71.86 (31.85)			
Mehmandoust (2024) DB-RCT in the community in Iran	*n* = 27 (15 male) mean (SD) age = 77.3 (8.4) mean (SEM) MMSE = 16.1 (5.4)	n = 25 (9 male) mean (SD) age = 77.0 (8.5) mean (SEM) MMSE = 16.6 (5.4)	*L. rhamnosus*, *Lactobacillus bulgaricus*, *L. casei*, *L. acidophilus*, *Bifidobacterium breve*, *B. longum* and *Streptococcus thermophilus* and FOS, 1× 10^9^ CFU for 12 weeks	Maltodextrin	MMSE: No significant difference, *P* = 0.53
Ni Lochlainn, (2024) DB-RCT in the community in the UK	*n* = 36 (8 male) mean (SD) age = 73.1 (4.9) mean (SD) CANTAB: Cognition Factor Score = 0.05 (1.00) OTS = 37476.2 (24711.6) Spatial working memory = 13.0 (8.7) PAL first attempt = 12.5 (4.5) PAL total errors = 16.7 (15.9) Pattern Recognition = 87.0 (11.7) Spatial span = 5.9 (1.6)	*n* = 36 (8 male) mean (SD) age = 73.1 (4.9) mean (SD) CANTAB: Cognition Factor score = 0.23 (0.94) OTS = 28766.9 (14250.0) Spatial working memory = 15.3 (11.0) PAL first attempt = 11.4 (4.7) PAL total errors = 19.2 (16.7) Pattern Recognition = 91.7 (9.0) Spatial span = 5.5 (1.0)	Inulin 3375 mg and FOS 3488 mg of per level measuring spoon (of 7.5 grams) for 12 weeks	Maltodextrin	CANTAB Cognition Factor Score: significant improvement, *P* = 0.014 Executive Function: no significant difference in OTS, *P* = 0.889, or Spatial Working Memory, *P* = 0.457 Memory: significant improvement in PAL total number of errors, *P* < 0.001; no significant difference in PAL first attempt (*P* = 0.104), Pattern Recognition (*P* = 0.331) or Spatial Span (*P* = 0.120)
Leblhuber (2018) Interventional study of outpatients in Austria	No control group	*n* = 20 (11 male) mean (SD) age = 76.7 (9.7) mean (SD) MMSE = 17.9 (7.9) CDT = 4.1 (2.8)	*L. casei* W56, *L. lactis* W19, *L. acidophilus* W22, *B. lactis* W52, *L. paracasei* W20, *L. plantarum* W62, *B. lactis* W51, *B. bifidum* W23 and *L. salivarius* W24, 4 weeks		MMSE: No significant difference

ADAS-Jcog = Alzheimer’s Disease Assessment Scale; CANTAB = Cambridge Neuropsychological Test Automated Battery; CDT = Clock Drawing Test; CERAD-K = Korean version of the Consortium to Establish a Registry for Alzheimer’s Disease; CFU = Colony Forming Units; DB-RCT = Double-Blind Randomised Controlled Trial; FOS = fructooligosaccharides; MMSE = Mini-Mental State Examination; MoCA = Montreal Cognitive Assessment; n = number; NA = not applicable; OTS=One Touch Stockings of Cambridge Cognitive Test; PAL = Paired Associates Learning; POCI = Post-Operative Cognitive Impairment; RBANS = Repeatable Battery for the Assessment of Neuropsychological Status; TMT = Trail Making Test (A and B); TYM = Test Your Memory; WLMIR = Word List Memory Immediate Recall; RCT = Randomised Controlled Trial; SD=Standard Deviation; SEM = Standard Error of the Mean; TMT = Trail Making Test (A and B); TYM = Test Your Memory; WLMIR = Word List Memory Immediate Recall.

Study settings included community, outpatient, hospital or nursing home care and sample sizes ranged from 40 to 120. Means ages of participants ranged from 64.5 to 82 years in the control group and 64.1 to 79.7 years in the intervention groups, with most participants being septuagenarians. Eleven studies reported baseline body mass index (BMI) that ranged from 21.7 to 28.8.

Six studies recruited cognitively healthy older adults [24–29], four recruited those with mild cognitive impairment [30–33] and four recruited those with dementia [23, 34–36].

### Intervention

The duration of interventions across studies ranged from short perioperative delivery to 24-week supplementation, with the modal timeframe being 12 weeks. Of 10 studies using probiotic interventions only, four employed single strain interventions while the remainder used muti-strain combinations, the greatest being 18 strains in a single intervention and the most commonly used bacterial genus being *Bifidobacterium*. Probiotic dosages and formulations ranged significantly from 10^7^ colony forming units (CFU) [29] to 5 × 10^10^ CFU [32], being administered as capsules, powdered or liquids. The interventional study did not state the concentration of probiotic sachets [23]. Both prebiotic studies used the same combination of inulin and fructooligosaccharides (FOS) [24, 27] and one synbiotic trial combined multiple bacterial genera with FOS [36], while the other administered a probiotic with and without freeze-dried berries [26].

### Cognitive outcomes

Cognitive outcomes were measured by validated neuropsychological assessments, most commonly the mini-mental state examination (MMSE), either in isolation or in combination with other assessment tools (see Table 1 for details). Nine studies reported statistically significant improvements in at least one cognitive domain, such as global cognition, memory, attention or executive function. Of the probiotic studies, six studies reported statistically significant improvements in global cognition [28, 29, 35] and/or sub-domains of attention, mental flexibility, recall, visuospatial and executive functions, memory and learning or verbal fluency [25, 28, 31, 32]. Two further probiotic studies did not find significant changes in global cognitive scores but demonstrated improvement in sub-domains visual memory (*P* = 0.044) [33] and orientation (*P* = 0.021) [30]. One probiotic study compared different dosages; the higher dose (10^8^ CFU) found a significant improvement in memory, learning process and verbal fluency (*P* < 0.05), whereas the lower dose (10^7^ CFU) reported significant improvement in memory, visuospatial, and verbal fluency (*P* < 0.05). The control group (placebo) recorded a significant improvement just in memory (*P* < 0.05) [32]. Improvement of the learning process occurred only in the higher dose group (*P* = 0.006).

One prebiotic study reported significant improvements in the CANTAB Cognition Battery via factor analysis (*P* = 0.014) and specifically in memory function (Paired Associates Learning total errors, *P* < 0.001) [27]. No significant changes in any domain were recorded by two probiotic trials [23, 34], one prebiotic trial (*P* = 0.85) [24] or either synbiotic trials (*P* = 0.53) [26, 36].

### Meta-analysis

Six RCTs used the MMSE as an outcome measure allowing a meta-analysis of this sub-group. The analysis demonstrated a statistically significant improvement (*P* < 0.05) in global cognition after supplementation compared with placebo, despite substantial heterogeneity (*k* = 6; MD = 1.28; 95% CI = 0.15–2.40; I^2^ = 71%; *Z* = 2.23, *P* = 0.03) (Figure 2A). A sensitivity analysis excluding moderate or high risk of bias trials yielded a comparable effect size, although again had high heterogeneity (*k* = 4; MD = 1.39; 95% CI = 0.03–2.75; I^2^ = 83%; *Z* = 2.00, *P* = 0.05) (Figure 2B). Standardised measurements showed a similar trend of a modest yet significant positive effect of supplementation (*k* = 6; SMD = 0.38; 95% CI = 0.02–0.74; I^2^ = 68%; *Z* = 2.05; *P* = 0.04), including when sensitivity analysis was conducted (*k* = 4; SMD = 0.52; 95% CI = −0.01-1.05; I^2^ = 79%; *Z* = 1.93; *P* = 0.05). These analyses provide a quantitative result consistent with the wider review findings of the benefits of supplementation.

**Figure 2:**
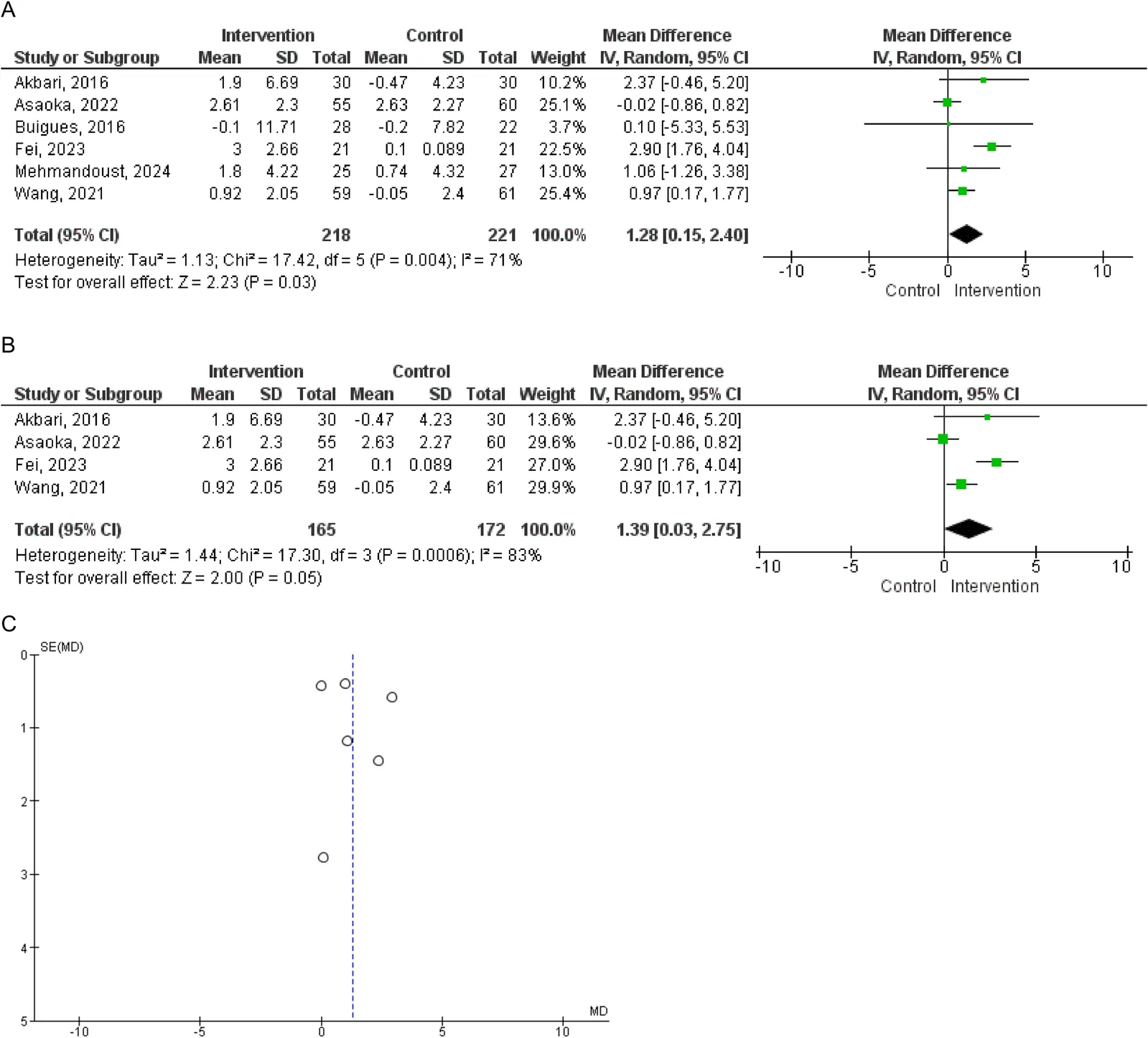
A) Sub-group analysis of effect of probiotics on global cognitive function as measured by MMSE (mean difference = 1.28; 95% confidence intervals = 0.15–2.40; I^2^ = 71%; overall effect *Z* = 2.23, *P* = 0.03); B) Sensitivity analysis of meta-analysis when moderate risk of bias trials removed; C) Funnel plot of trials included in the meta-analysis.

### Secondary outcomes

Several studies also reported secondary outcomes beyond cognitive function (Table 2), including gut microbiome composition, sleep, mood, physical function and inflammatory markers. Eight of the studies reported gut microbiome composition and any relevant changes. While Fei et al. were the only trial to report a significant change in beta diversity [31], the remaining probiotic trials reported increases in beneficial taxa, mainly at the genus level, such as *Bifidobacterium* and *Lachnospira* and reductions in pro-inflammatory genera, including *Collinsella* and *Parabacteroides* [25, 28, 29, 31, 33]. Improvements in inflammatory markers, lipid profiles, sleep quality, mood and physical function were reported, particularly in multi-strain interventions [25, 26, 29, 31, 35]. One prebiotic intervention showed significantly improved frailty indices, particularly reducing exhaustion and improving muscle function and handgrip strength [24].

**Table 2:** Secondary outcomes.

Study	Change in gut microbiome composition	Health benefits
Akbari (2016)	Not tested	Significant positive effects on inflammatory markers MDA and CRP, insulin metabolism markers, triglyceride and VLDL serum level. No changes were recorded in other lipid profiles or biomarkers of inflammation or oxidative stress.
Buigues (2016)	Not tested	Significant improvement in exhaustion/fatigue and grip strength in the dominant hand. No significant improvements in other frailty criteria, metabolic electrolyte or inflammatory markers.
Agahi (2018)	Not tested	No significant changes in inflammatory or antioxidant markers
Kim (2021)	No significant changes in diversity or at phylum level but relative abundances of pro-inflammatory genera Eubacterium, Allisonella, Clostidiales and Prevotellaceae decreased	Significant improvements in flatulence frequency, abdominal distension, stress scores and BDNF, which was also negatively correlated with Eubacterium and Clostridiales
Wang (2021)	Faecal microbiome measurements found no significant change at phylum level but increases at genus level in Eggerthella, Actinomyces, Enterococcus, Lactobacillus, Megaspaera, Lachnospiraceae and Steptococcus. (Control group recorded increases in Enterococcus, Ruminococcus and Ruminococcaceae).	Significant decrease in post-operative IL-6, cortisol. No changes in pain intensity, sleep quality or GI function recovery.
Asaoka (2022)	No significant change in GM composition due to supplementation. The lower sub-group of baseline MMSE scores had lower relative abundances of the phylum Actinobacteria and the genus Bifidobacterium and higher abundances of Prevotella, Clostridiaccae, Ruminococcaceae and Phascolarctobacterium.	Significant difference in grey matter atrophy for the whole brain
Handajani (2022)	Not tested	Reported delivery of lactic acid bacterial isolates carrying a glutamate decarboxylase coding genes that are involved in the synthesis of the neurotransmitter, GABA
Sakurai (2022)	No significant change in alpha or beta diversity. Significant increase in relative abundances of genera Lachnoclostridium, Monoglobus and Oscillibacter	Not measured
Fei (2023)	Significant difference in beta diversity between groups with increased abundance of phylum Bacteroidetes and genera Blautia, Lachnospiraceae, Muribaculaceae, Haemophilus, Coprococcus, Ruminococcus, Anaerostipes, Erysipelotrichaceae, Prevotellaceae and Pantoea	Significant improvement in overall sleep quality and GI symptom scores and serum BDNF
Shi (2023)	No significant change to alpha or beta diversity of the GM. At phylum level: increase in relative abundance of Firmicutes and Actinobacteria with a decrease in Proteobacteria. At genus level: significant increase in relative abundances of Bifidobacterium, Dorea, Lachnospira and Cellulosilyticum and reduction in Solobacteria, Oribacterium, Eubacterium, Collinsella, Parabacteroides, Tyzzerella, Bilophila and Negativicutes. Enriched taxa in control group: Clostridium, Cillinsella, Epulopiscium, Porphyrimonas, Negativicutes and Granulicatella.	Not measured
Lazou-Ahren (2024)	Not tested	Significant decrease in faecal calprotectin levels and reduction in bodily pain. No difference in CRP, fibrinogren, total serum protein, faecal zonulin, any other measured inflammatory markers, frequency of motions, stool consistency, liver or kidney function. Placebo group recorded a significant reduction in systolic blood pressure and probiotic group had a significant reduction in social functioning.
Mehmandoust (2024)	Not tested	No significant changes in FBG, lipid profiles, serum albumin or vitamin D levels.
Ni Lochlainn (2024)	No significant differences in alpha or beta diversity. Significant increase in abundance of Bifidobacterium from phylum level of Actinobacteria to genus, with smaller increases in Firmicutes and Bacteroidetes and a decrease in genera Phocea and Anaeromassilibacillus, order Desulfovibrionales, Lactobacillales, Acidamincoccales, Eubacteriales	No significant improvement in muscle strength and function or appetite
Leblhuber (2018)	Faecal microbiome showed significant increase in *Faecalibacterium prausnitzii* and decrease in zonulin.	Significant increase in kynurenine serum levels but did not affect the kynurenine/tryptophan ratio

BDNF = brain-derived neurotropic factor; CRP = C-reactive Protein; FBG = Fasting Blood Glucose; GABA = Gamma-Aminobutyric Acid; GI = Gastrointestinal; IL-6 = Interleukin 6; MDA = Malondialdehyde; VLDL = Very-Low-Density Lipoprotein.

### Risk of bias

Most RCTs were judged to be low risk of bias due to blinding and randomisation processes (Figure 3A). Handajani et al. [32] was rated at high risk due to incomplete outcome data and unclear allocation methods, while six studies showed some concern [24, 26, 28, 33, 34, 36]. The single interventional study was deemed as critical risk of bias (Figure 3B), driven by potential confounding and participant selection [23].

**Figure 3:**
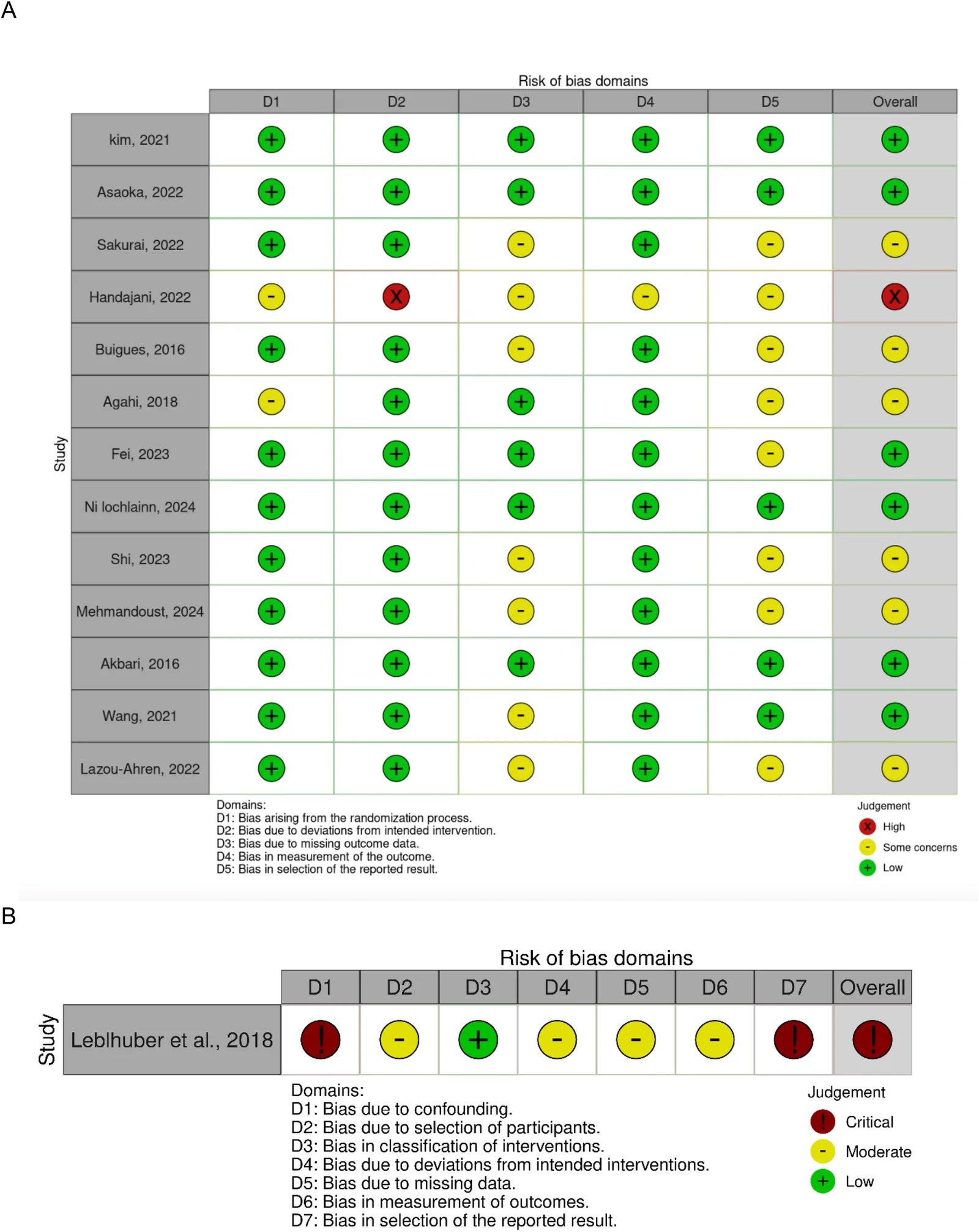
A) Risk of bias assessments on RCTs using Cochrane RoB2; B) Risk of bias assessment on the interventional study using Cochrane ROBINS-I tool; displayed as a traffic light plot.

The overall risk of bias judgements were incorporated into the GRADE assessments (See: Appendices, Supplementary material), which indicated low to moderate certainty of evidence across different cognitive outcomes such as global cognitive function and memory performance. Certainty was downgraded due to inconsistency and imprecision reflecting the small sample sizes, short study duration and differences in size and measurements of outcomes. Funnel plot asymmetry (Figure 2C) is suggestive of publication bias, although there were too few studies (*k* < 10) for formal assessment with Egger’s or Begg’s test [37].

### Sensitivity analysis

Qualitative sensitivity analyses were conducted by systematically excluding studies based on key criteria and examining the impact on overall results. Excluding studies with elevated risk of bias maintained a consistent pattern of cognitive benefit [25, 27, 29–31, 35], while restricting analyses to participants without advanced cognitive impairment (defined as a baseline MMSE score of <23) showed cognitive improvements, especially in memory and executive function. An analysis of just the probiotic trials similarly resulted in positive cognitive outcomes, mainly in memory and mental flexibility [23, 25, 28–35], with some evidence of neuroprotective effects such as reduced brain atrophy or reduced incidence of postoperative cognitive impairment [29, 30].

## Discussion

This systematic review and meta-analysis synthesised evidence from 14 studies evaluating the effects of prebiotic, probiotic and synbiotic interventions on cognitive function in adults aged ≥60 years. Despite heterogeneity in populations, interventions and outcome measures, the overall direction of effect consistently favoured the intervention groups with improvements observed across multiple cognitive domains. The meta-analysis of MMSE outcomes demonstrated a statistically significant, though modest, improvement in global cognition. The substantial heterogeneity across pooled studies limits its precision as a definitive treatment but acts as supportive evidence, estimating an average positive impact. These findings show that gut microbiome modulation delivers measurable improvements and supports gut microbiome-targeted interventions as potential adjuncts to cognitive ageing.

The most consistent cognitive improvements were observed in measures of global cognition and in cognitively healthy or mildly impaired participants. These outcomes suggest that gut microbiome modulation may influence broader cognitive functioning and may be relevant to slowing early decline or maintaining cognitive function. This is supported by the small-to-moderate effect size (SMD = 0.38), which illustrates consistent and reproducible benefit but likely represents limited patient-reported clinical gains, especially in advanced impairment. A preventative strategy is often preferrable to managing established conditions. In the context of ageing, even modest stabilisation of cognition may prolong independence and improve quality of life. Thus, even studies which show an improvement in one cognitive domain can be taken as a positive result to inform futures RCTs. More work is needed to establish whether cognitive improvements are clinically meaningful.

Probiotic interventions accounted for most included studies (10/14) and demonstrated the most consistent cognitive effects, especially those involving multi-strain, high-dose formulations. This pattern likely reflects the wide variety of health benefits of gut microbiome modulation, with significant clinical implications for the older population. Probiotic and prebiotic supplementation has been shown to strengthen the gut barrier and reduce chronic inflammation associated with neurodegenerative and vascular ageing processes [38, 39]. Common probiotic genera, *Bifidobacterium* and *Lactobacillus,* are involved with neurotransmitter synthesis and gut-brain axis signalling pathways [40, 41] which aligns with their positive associations in this review. Specifically, a probiotic trial showing suppression of global grey matter atrophy alongside trials showing increases in brain-derived neurotrophic factor, correlated with reductions of pro-inflammatory genera *Eubacterium* and *Clostridiales*, as well as lactic acid bacteria supplements carrying GABA synthesis genes, suggests direct microbial influence on cognitive maintenance [25, 30, 31]. The diverse and complementary effects of gut microbiome modulators may be why high-dose and multi-strain supplements result in beneficial outcomes. Although probiotic formulations differed significantly between interventions, the shared treatment strategy allowed pooling for comparisons and identification of a class effect. However, variation of probiotic effects based on dose and strain means that not all interventions should be considered equivalent and specific treatment recommendations cannot yet be made. Additionally, the range of formulations and small number of prebiotic-only and synbiotic interventions (two studies of each) preclude meaningful comparisons between intervention types.

The observed cognitive benefits may point towards broader systemic health benefits delivered by microbial metabolites, including improvements to metabolic and immune function in older adults [42]. Some studies in this review reported reduction of pro-inflammatory markers, such as interleukin-6 and faecal calprotectin, as well as improvements in insulin resistance and triglyceride levels [26, 29, 35]. In terms of daily physical functioning, gut microbiome modulation has been shown to support muscle mass and strength [43], as well as playing a potential role in maintaining bone health of post-menopausal women [44]. The variety of actions by gut microbiome-targeted interventions raises the possibility that cognition also benefits indirectly as part of a holistic health effect, including improvement in sleep quality, reducing gastrointestinal symptoms and exhaustion [24, 25, 31]. Whilst not all mechanisms are fully understood, from a clinical perspective, the gut microbiome potentially represents a single, non-pharmacological, low-risk, implementable strategy to mitigate age-associated morbidity. With the high adherence found in the selected studies suggesting these interventions are well tolerated and many of these supplements already being commercially available, it may offer a relatively cheap and scalable intervention to support public health and potentially relieve pressures on the healthcare systems by an ageing population.

Study durations were generally short (mode = 12 weeks) with none including long term follow-up. Several studies demonstrated cognitive improvements within this brief timeframe, suggesting that gut microbiome-targeted interventions may confer benefits relatively quickly. However, the absence of longitudinal data limits conclusions over the durability of these effects and whether cognitive improvements translate to sustained clinically meaningful benefits. These findings should be interpreted as short term effects and trials with extended follow-up are warranted to determine the longevity or transience and clinical significance of these early effects.

### Strengths, limitations and quality of evidence

This review is strengthened by strict adherence to a predefined protocol and PRISMA guidance, with independent screening and use of standardised assessments tools for risk of bias (RoB2/ROBINS-I) and quality of evidence (GRADE). These methodological measures minimise reviewer bias, providing objectivity, transparency and reproducibility of results. Furthermore, sensitivity analyses demonstrated the overall pattern of cognitive outcomes was not due to studies judged to be at high risk of bias, supporting the robustness of the observations made.

Strengths of the literature include uniformity of oral intervention delivery, the studies were conducted in wide variety of clinical and geographic locations across Asia, the Middle East and Europe, and participants ranged from the cognitively healthy to those with Alzheimer's disease. This diversity enhances the external validity of findings and provides a more globally representative picture of supplementation effects in different clinical and community settings.

The lack of uniformly reported baseline diets, nutritional statuses and gut microbiome compositions act as potential cofounders. Diet is key a determinant of gut microbiome composition and older adults are at higher risk of malnutrition for a variety of biological, psychological and social reasons. Therefore, an unbalanced or nutritionally replete diet will likely impact the diversity and health of the gut microbiome. Without consistent control or reporting of baseline diets, unknown factors may influence the true effect of interventions. Additionally, with an incomplete record of how each intervention affected the gut microbiome composition, it is challenging to identify which specific bacterial taxa are associated with cognitive changes and map the biological mechanisms of the gut-brain axis. Furthermore, baseline cognitive status varied across populations of the included studies. Sensitivity analyses confirmed consistent benefits in cognitively healthy and mildly impaired populations, whereas results were more mixed in those with dementia. These groups may differ in underlying disease pathology and responsiveness to short term gut microbiome-targeted interventions according to baseline cognitive status. Therefore, pooled findings should be interpreted cautiously, and future trials should stratify participants accordingly as benefits may not be equivalent across all cognitive groups.

Limitations of the literature additionally include imprecision and inconsistent reporting of randomisation, adherence and outcomes. However, the common use of the MMSE (7/14 trials, 6/13 RCTs) advantageously allowed for sub-group meta-analysis, providing a quantitative justification for our conclusions. A low-quality non-RCT was included as it aligned with the inclusion criteria and contributed to qualitative synthesis of results due to the limited volume of available literature, but data could not be included for meta-analysis. Conservative estimation of variance measures in some cases potentially introduces additional uncertainty, yet the overall direction of effect consistently favoured intervention groups, supporting the use of supplementation. Grey literature and non-English studies were not systematically searched; publication and language bias cannot be excluded for missing wider literature. These limitations were considered when appraising the certainty of evidence and drawing overall conclusions.

## Conclusion

This systematic review and meta-analysis present evidence that probiotic, prebiotic or synbiotic supplementation yields modest but measurable cognitive improvements in adults over 60 years, particularly in global cognition, memory and executive functions. These findings support gut microbiome modulation as a potential affordable, non-invasive strategy to promote cognitive health, as well as holistic healthy ageing due to its wider benefits.

Current evidence remains limited, preventing recommendation of specific formulations or its use in routine clinical practice. However, gut microbiome-targeted interventions can be considered as low-risk adjuncts within a framework of healthy ageing, with potential value in early cognitive vulnerability and more research is needed in the setting of established disease. Future research should prioritise larger scale, longer-term trials with greater methodological standardisation, consistency in cognitive outcome measures and integration of lifestyle factors, especially diet. Determining optimal strain combination, doses and treatment durations is essential to maximise benefits across a diverse and ageing population. Strengthening this evidence base will help establish whether the observed effects can be translated into a sustained and clinically meaningful intervention.

## Supplementary Material

aa-26-0655-File002_afag287

aa-26-0655-File003_afag287

## Notes

PROSPERO Registration: CRD420251029045
