# Supplementary material for: Gut microbiome modulation can improve cognitive function in older adults: a systematic review and meta-analysis
Source: Age Ageing. 2026 Sep 29;55(9):afag287. doi: 10.1093/ageing/afag287 (PMC13619334; doi:10.1093/ageing/afag287)
Supplement: Supplementary file 1 [file aa-26-0655-file002_afag287.docx]

**Title: Gut Microbiome Modulation Can Improve Cognitive Function in Older Adults: A Systematic Review and Meta-Analysis**

**Supplementary Files**

**Contents**

Appendix 1. Databases and Search Strategy

Appendix 2. Inclusion/Exclusion Criteria

Appendix 3. Summary of findings table including GRADE assessments of certainty of evidence

**Appendix 1: Databases and Search Strategy**

| Database | Search Strategy | Number of Articles |
| --- | --- | --- |
| Pubmed | ((gut microbiome OR gut microbiota OR gut flora OR gut bacteria OR intestinal microbiome OR intestinal microbiota OR intestinal flora OR intestinal bacteria) AND (cognitive function OR cognitive performance OR cognitive decline OR cognitive impairment) AND (prebiotics OR probiotics OR synbiotics OR live bacteria OR microbial supplements OR prebiotic-probiotic combination) AND (geriatrics OR aged OR aging population OR seniors OR older adults OR elderly)) | 194 |
| Cochrane Library | ((gut microbiome OR gut microbiota OR gut flora OR gut bacteria OR intestinal microbiome OR intestinal microbiota OR intestinal flora OR intestinal bacteria) AND (cognitive function OR cognitive performance OR cognitive decline OR cognitive impairment) AND (prebiotics OR probiotics OR synbiotics OR live bacteria OR microbial supplements OR prebiotic-probiotic combination) AND (geriatrics OR aged OR aging population OR seniors OR older adults OR elderly)) | 126 |
| Medline + Embase (OVID) | ((gut microbiome or gut microbiota or gut flora or gut bacteria or intestinal microbiome or intestinal microbiota or intestinal flora or intestinal bacteria) and (cognitive function or cognitive performance or cognitive decline or cognitive impairment or mental health or mood) and (prebiotics or probiotics or synbiotics or live bacteria or microbial supplements or prebiotic-probiotic combination or dietary supplements or live microbes) and (geriatrics or aged or aging population or seniors or older adults or elderly)) | 3103 |

**Appendix 2: Inclusion/Exclusion Criteria**

| **Study characteristic** | **Inclusion Criteria** | **Exclusion Criteria** |
| --- | --- | --- |
| Population | Adults ≥ 60 years irrespective of health status | Participants ≤ 60 years |
| Intervention | Probiotics, Prebiotics or Synbiotics supplementation | Faecal transplants |
| Comparator | Placebo or control | Active comparators |
| Outcome | Primary outcome: changes in cognition e.g. memory, attention, executive function or overall cognitive performance  Secondary outcome (where reported): other health effects e.g. gut microbiome composition, changes in physical function | Self-reported measures |
| Outcome Measure | Validated neuropsychological tools or standardised cognitive tests e.g. MMSE, MoCA | Lacking clear cognitive outcome assessments |
| Study Design | RCT and observational studies | Qualitative studies |
| Publication | Completed studies reporting original research results, published in peer-review journals | Duplicates, reviews, unpublished as full text, retracted articles or in vivo/vitro experiments |

MMSE = Mini-Mental State Examination; MoCA = Montreal Cognitive Assessment; RCT = Randomised Control Trials

**Appendix 3: Summary of findings table including GRADE assessments of certainty of evidence**
